# Unique *N*-glycosylation of a recombinant exo-inulinase from *Kluyveromyces cicerisporus* and its effect on enzymatic activity and thermostability

**DOI:** 10.1186/s13036-019-0215-y

**Published:** 2019-10-29

**Authors:** Junyan Ma, Qian Li, Haidong Tan, Hao Jiang, Kuikui Li, Lihua Zhang, Quan Shi, Heng Yin

**Affiliations:** 1 Natural Products and Glyco-Biotechnology Research Group, Liaoning Province Key Laboratory of Carbohydrates, Dalian Institute of Chemical Physics, Chinese Academy of Sciences, Dalian, 116023 China; 2 Liaoning Province Key Laboratory of Bio-Organic Chemistry, Dalian University, Dalian, 116622 China; 3 Dalian Institute of Chemical Physics, Chinese Academy of Sciences, Dalian, 116023 China

**Keywords:** *N*-glycosylation, Exo-inulinase, *β*-Sandwich domain, Enzyme activity, Thermostability

## Abstract

**Background:**

Inulinase can hydrolyze polyfructan into high-fructose syrups and fructoligosaccharides, which are widely used in food, the medical industry and the biorefinery of *Jerusalem artichoke*. In the present study, a recombinant exo-inulinase (rKcINU1), derived from *Kluyveromyces cicerisporus* CBS4857, was proven as an *N*-linked glycoprotein, and the removal of *N*-linked glycan chains led to reduced activity.

**Results:**

Five *N*-glycosylation sites with variable high mannose-type oligosaccharides (Man_3–9_GlcNAc_2_) were confirmed in the rKcINU1. The structural modeling showed that all five glycosylation sites (Asn-362, Asn-370, Asn-399, Asn-467 and Asn-526) were located at the C-terminus *β*-sandwich domain, which has been proven to be more conducive to the occurrence of glycosylation modification than the N-terminus domain. Single-site *N*-glycosylation mutants with Asn substituted by Gln were obtained, and the Mut with all five *N*-glycosylation sites removed was constructed, which resulted in the loss of all enzyme activity. Interestingly, the N362Q led to an 18% increase in the specific activity against inulin, while a significant decrease in thermostability (2.91 °C decrease in *T*_*m*_) occurred, and other single mutations resulted in the decrease in the specific activity to various extents, among which N467Q demonstrated the lowest enzyme activity.

**Conclusion:**

The increased enzyme activity in N362Q, combined with thermostability testing, 3D modeling, kinetics data and secondary structure analysis, implied that the *N*-linked glycan chains at the Asn-362 position functioned negatively, mainly as a type of steric hindrance toward its adjacent *N-*glycans to bring rigidity. Meanwhile, the *N*-glycosylation at the other four sites positively regulated enzyme activity caused by altered substrate affinity by means of fine-tuning the *β*-sandwich domain configuration. This may have facilitated the capture and transfer of substrates to the enzyme active cavity, in a manner quite similar to that of carbohydrate binding modules (CBMs), i.e. the chains endowed the *β*-sandwich domain with the functions of CBM. This study discovered a unique C-terminal sequence which is more favorable to glycosylation, thereby casting a novel view for glycoengineering of enzymes from fungi via redesigning the amino acid sequence at the C-terminal domain, so as to optimize the enzymatic properties.

## Background

Inulinases can hydrolyze *β*-2,1-D-fructosidic linkages in polyfructan, which are found in a wide range of plants and microorganisms, among which yeasts, especially *Kluyveromyces*, generally produces higher levels of inulinase than other filamentous fungi or bacterial [1]. The hydrolysis products, high-fructose syrups and fructoligosaccharides, are widely used in food, medical and bioenergy industries. Inulinases are divided into endo-inulinase (EC 3.2.1.7) and exo-inulinase (EC 3.2.1.80) according to the mode of action on inulin [2]. Exo-inulinase as the key enzyme in *Jerusalem artichoke* biorefinery can hydrolyze inulin from the non-reducing end, obtaining the high-fructose syrups with purity up to 95% within one step, which can be further transformed into fuel ethanol, single cell oil, 2,3-butanediol, lactic acid or other chemical production [3]. Therefore, exo-inulinase has attracted much attention in scientific research for its potential application in biomass processing.

Exo-inulinases belong to the family of glycoside hydrolase 32 (GH32), a family including other inulinases, invertases, *β*-fructofuranosidases and fructosyltransferases, which share a common structural feature: a N-terminal five-blade *β*-propeller catalytic domain with four antiparallel *β*-strands exhibiting ‘W’ topology for each blade, surrounding the catalytic active center, followed by a C-terminal *β*-sandwich domain constituting with two antiparallel six-stranded *β*-sheets [4]. At present, though with much more penetration in the research about the N-terminal catalytic module, little is known about the function of the C-terminal domain. However, researchers found that it might be involved in substrate binding [5, 6] and regulating substrate specificity [7]. For example, the *β*-sandwich domain of a dimeric *β*-fructofuranosidase from *Schwanniomyces occidentalis* was found to be involved in substrate binding through the interaction between *β*-sandwich domain of the adjacent subunit within the dimer with the substrate [6]; and the C-terminal domain (BsCBM66) of the exo-acting levanase SacC from *Bacillus subtilis* proved to belong to CBM66 with the function of identifying and binding substrate, as well as facilitating the orienting of the catalytic domain to the substrate [8, 9], thereby enhancing enzymatic activity through increasing the concentration of the appended enzymes in the vicinity of the substrate. It was just the extensive interactions of BsCBM66 with the terminal fructose moiety (Fru-3) of levantriose that conferred SacC the substrate specificity [7].

In recent years, many exo-inulinases were cloned in various hosts and characterized [2, 10–12], and it was found that exo-inulinases expressed by yeast strains, such as *P. pastoris*, were usually modified by *N*-linked glycosylation [1, 13], one of the most common and important post-translational modifications in eukaryotic cells. *N*-glycosylation often affects the folding and conformation of proteins [14, 15], secretory expression [16–18], enzyme activity [19] and stability [20]. The effects of *N*-glycosylation modification on the enzyme activity are quite complicated, which are including glycosylation sites, glycoforms and the length of glycan chains, etc. Glycosylation at different sites sometimes could lead to opposite effects on enzyme activity. As for Cellobiohydrolases (CBHs) PvCel6A and PvCel7A, both from *Penicillium verruculosum* belonging to GH6 [21] and GH7 family [22] respectively, the removal of *N*-linked glycans close to the entrance to the enzyme active center turned out to be an increase in the rate of cellulose hydrolysis likely due to the elimination of steric hindrance and the ease of threading the cellulose molecule into the tunnel. Meanwhile the deletion of *N*-linked glycans at the positions locating at the “bottom” of their catalytic domains resulted in a partial decrease of enzyme activity, because the glycan chains attaching to these residues could help the enzyme orient appropriately to the surface of cellulose microfibril. In most cases, the loss of *N*-linked glycosylation would reduce the enzyme activity, which was probably caused by the change of secondary structure via circular dichroism spectroscopy analysis [23]. Meanwhile it has been reported that the enzyme activity is increased by introducing glycosylation sites recently [18, 24]. In addition, enzymes which are from different expression systems often bring different activities due to variable glycoforms or the length of glycan chains. Wei et al. have reported that the hydrolytic activity of *Aspergillus terreus β*-glucosidase expressed by *P.pastoris* was lower than that by *Trichoderma reesei*, with a conclusion that longer *N*-linked glycan chains could weaken the substrate affinity [25].

In general, *N*-glycosylation plays diverse roles in regulating enzyme activity, though its mechanism is still not completely understood. The impact of *N*-glycosylation on the conformation of enzyme [14, 26–28] and the substrate affinity [25, 29] are probably the main factors for the changed activity. Gao et al. raised the hypothesis of “synergism” based on the research on CBHI from *Penicillium decumbens*, i.e. *N*-glycosylation of CBHI-A, which has no detectable hydrolysis activity, could favor for other cellulases to hydrolyze substrates due to the disruption of the hydrogen bonds from cellulose and the convertion of the high crystalline cellulose into amorphous types [30]. In addition, Dotsenko and Gusakov et al. pointed out the novel concept of “lifting factor” for the function of *N*-linked glycosylation [22]. They believed *N*-glycosylation located at the “bottom” of the catalytic domains of CBHs belonging to GH6 and GH7 families could help the catalytic domain of the enzymes positioning along the microcrystalline chains through non-specific dynamic interaction, followed by the detachment of a single cellulose chain from the crystal, i.e. the *N*-linked glycans served as a chain “lifting actor”, and then threaded substrates into the active tunnel.

However, little is known about the effects of *N*-glycosylation on exo-inulinase activity compared to the cellulose, and still with no report about the effect of *N*-glycosylation on the activity of inulinase at present. For GH32 family, limited research has reported about the influence of *N*-glycosylation on substrate specificity of fructan 1-exohydrolase [31], invertase activity or stability [32, 33] and *β*-fructofuranosidase activity as well as oligomerization [34].

In the present study, the recombinant *N*-glycoprotein rKcINU1 was expressed in *P. pastoris* X-33. Meanwhile single-site glycosylation mutants and the Mut with five *N*-glycosylation sites removed were constructed to investigate the effect of *N*-glycans on enzymatic activity, kinetics properties, the second structure and thermostability. Specifically, its five *N*-glycosylation sites were found to be located at the C-terminal *β*-sandwich domain and likelihood mechanism was given based on the experimental results and 3D modeling, which was the *N*-linked glycan chains endowed the *β*-sandwich region with the functions of CBM.

## Materials and methods

### Strains, plasmids and culture conditions

*Escherichia coli* TOP10 (TaKaRa, Dalian, China) was used as the host strain for plasmid amplification. *P. pastoris* X-33 (Invitrogen, Carlsbad, USA) served as the eukaryotic expression host for different enzymes. The plasmid pPICZαA (Invitrogen, Carlsbad, USA) was used to construct expression vectors. *E. coli* strain was cultivated in low salt Luria-Bertani (LB) medium containing 25 μg/mL zeocin at 37 °C. *P. pastoris* was grown in YPDSZ medium (w/v): 1% yeast extract, 2% peptone, 2% glucose, 1 M sorbitol and 100 μg/mL zeocin at 30 °C for selecting transformants. Protein expression was performed in BMGY and BMMY media (w/v) at 28 °C: 1% yeast extract, 2% peptone, 1.34% YNB, 4 × 10^− 5^% biotin, 100 mM potassium phosphate, pH 6.0 and 1% glycerol or 0.5% methanol as carbon sources, respectively.

### Site-directed mutagenesis, protein expression and purification

The exo-inulinase gene *kcINU1* from *K. cicerisporus* CBS4857 (GenBank™ accession number AF178979) was amplified and cloned into the pPICZαA expression vector to yield the plasmid pPICZαA-rkcINU1 as described previously [35]. Restriction-free cloning was carried out to construct the *N*-glycosylation mutations N362Q, N370Q, N399Q, N467Q and N526Q. The Asn codons were mutated to Gln using specific primers listed in Additional file 1: Table S1 with pPICZαA-rkcINU1 as a template following the method published previously [35]. A variant designated as Mut with all five glycosylated Asn sites mutated into Gln was also obtained. The protein expression and purification followed the method described by Ma [35], with details described in Additional file 1.

### Glycosidase treatment, Zymogram analysis and enzymes activity assays

The deglycosylation reaction of purified wild-type rKcINU1 using PNGase F (New England Biolabs, Beverly, MA, USA) and EndoF1 (Sigma, Saint Louis, MO, USA) were carried out according to the specification. The zymogram analysis and activity assay by DNS method were described as referred previously [35]. The details above were given in Additional file 1.

### Kinetics parameters

For kinetics study, the initial velocities were measured by incubating 0.4 μg of purified rKcINU1 and its variants with 1–20 mg/mL sucrose at 55 °C for 20 min in 100 mM acetate buffer, pH 4.5. The initial rates versus substrate concentrations were hyperbola fitted based on the Michaelis-Menten equation using Origin program (version 8). Kinetics parameters *K*_*m*_ and *V*_*max*_ were calculated from the curve.

### Mass spectrometry analysis

Glycosylation sites of the purified wild-type rKcINU1 and Mut were confirmed by using the mass spectrometry analysis described previously by Jiang et al. [36]. The details involved were given in Additional file 1.

### Three-dimensional modeling

The three-dimensional structure of the wild-type rKcINU1 was built by the Swiss-Model server (http://swissmodel.expasy.org/) based on the crystal structure of *S.cerevisiae* invertase (PDB code 4EQV) which sharing 54.82% sequence identity with the rKcINU1. Docking of the *N*-linked glycan chains to the model mentioned above was then followed using the program GLYCAM, whereas the (Man)_7_(GlcNAc)_2_ glycan chains were selected from the high-mannose oligosaccharide library.

### Circular Dichroism (CD) spectroscopy analysis

Far-UV CD spectra of the purified wild-type rKcINU1 and its mutants were measured in 20 mM phosphoric acid (pH 4.5) at a protein concentration of 0.2 mg/mL with a MOS-450 (Bio-Logic, Seyssinet-Pariset, France) spectropolarimeter using 2 mm path length quartz cuvettes. The protein spectra were recorded over a wavelength range from 190 nm to 260 nm at 25 °C with a 1 nm bandwidth. The speed of scanning was 50 nm/min. For each sample, the average of three spectra was collected by subtraction of the equivalent buffer spectrum. The secondary structure of each sample was analyzed with CDpro software.

### Differential scanning Calorimetry (DSC) analysis

The thermodynamic parameters of wild-type rKcINU1 and its variants were determined on a Nano-Differential Scanning Calorimeter (Nano-DSC, TA Instruments, New Castle, DE, USA). The purified recombinants mentioned above were prepared in 100 mM sodium acetate buffer at pH 4.5 with a final protein concentration of 1 mg/mL. The samples were heated in the temperature ranging from 25 °C to 110 °C with the acceleration rate of 1 °C/min. All samples were degassed in vacuum prior to subjecting to the system, with a baseline of 100 mM sodium acetate buffer, pH 4.5. The protein spectra were recorded by subtraction of the baseline. Data analysis was performed on the DSC run software.

## Results and discussion

### The rKcINU1 modified by *N*-glycosylation

**Fig. 1:**
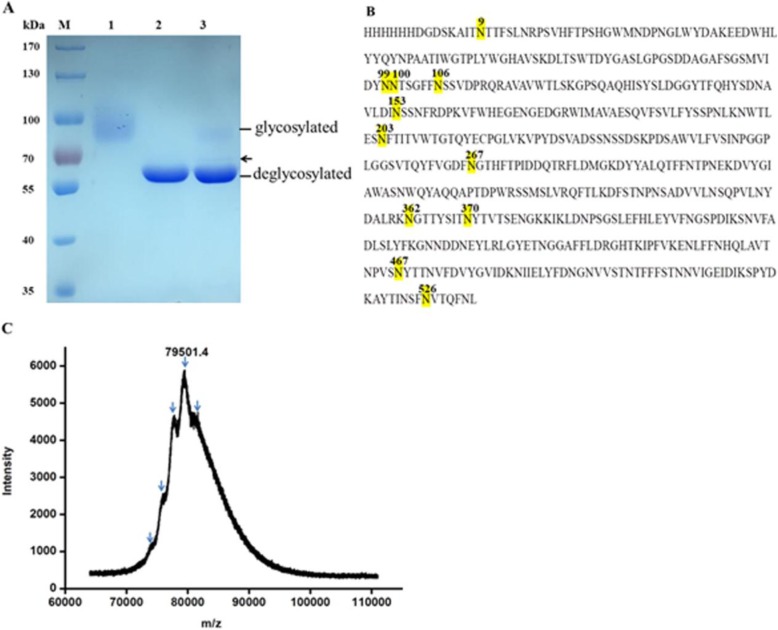
Analysis of *N*-glycosylation modification of the exo-inulinase from *K. cicerisporus* CBS4857 expressed in *P. pastoris* X-33. **a**, SDS-PAGE analysis of purified rKcINU1 treated with PNGase F. Lane M: the protein maker; Lane 1: the purified rKcINU1; Lane 2: the purified rKcINU1 treated with PNGase F under the denaturing condition; Lane 3: the purified rKcINU1 treated with PNGase F under the natural condition. **b**, The amino acid sequence of rKcINU1 with the putative *N*-glycosylation sites. The Glu just following the polyhistidines at the *N*-terminus was designated as the first amino acid site for the mature enzyme. Eleven potential glycosylation sites were shown in yellow including Asn-9, Asn-99, Asn-100, Asn-106, Asn-153, Asn-203, Asn-267, Asn-362, Asn-370, Asn-467 and Asn-526. **c**, The determination of molecular mass of rKcINU1 by MALDI-TOF mass spectrometry. The arrows indicated different glycoform ensembles with variable molecular mass

The recombinant rKcINU1, which corresponds to the mature enzyme sequence with an additional N-terminal His-tag, consists of 538 amino acid residues, with its theoretical molecular mass deduced from its primary sequence as 60.54 kDa. However, the band of purified rKcINU1 smeared between 70 kDa and 100 kDa (Fig. 1 a, Lane 1), which is much higher than the calculated mass of 60.54 kDa. This is likely due to the glycosylation modification of the enzyme by *P. pastoris*, which has the ability to post-translationally modify recombinant proteins [1, 37]. An analysis of the amino acid sequence of rKcINU1 produced 1 potential *N*-glycosylation sites using the website named NetNGlyc 1.0 Server (http://www.cbs.dtu.dk/services/NetNGlyc/), which were then used to predict the *N*-glycosylation sites (Fig. 1 b). Next, rKcINU1 was digested with PNGase F, which was known to cleave *N*-linked glycans between the innermost acetylglucosamine (GlcNAc) and the Asn residue, a single band instead of smear appeared in the SDS-PAGE (Fig. 1 a, Lane 2), which was almost exactly consistent with the theoretical molecular weight of 60.54 kDa. Later, MALDI-TOF mass spectrometry was used to obtain the precise molecular mass of rKcINU1 79.5 kDa (Fig. 1 c). All of the data indicated that rKcINU1 was an *N*-glycoprotein, and its average degree of glycosylation was 23.9%. Furthermore, rKcINU1 was digested with PNGase F under natural conditions (Fig. 1 a, Lane 3), and the results showed a band of about 65 kDa on the gel, hinting at the fact that rKcINU1 possessed more than one glycosylation site, some of which were likely buried deep inside the enzyme, where they inhibited the accessibility of PNGase F to those sites.

### Effect of Deglycosylation by Endo F1 on enzyme activity

**Fig. 2:**
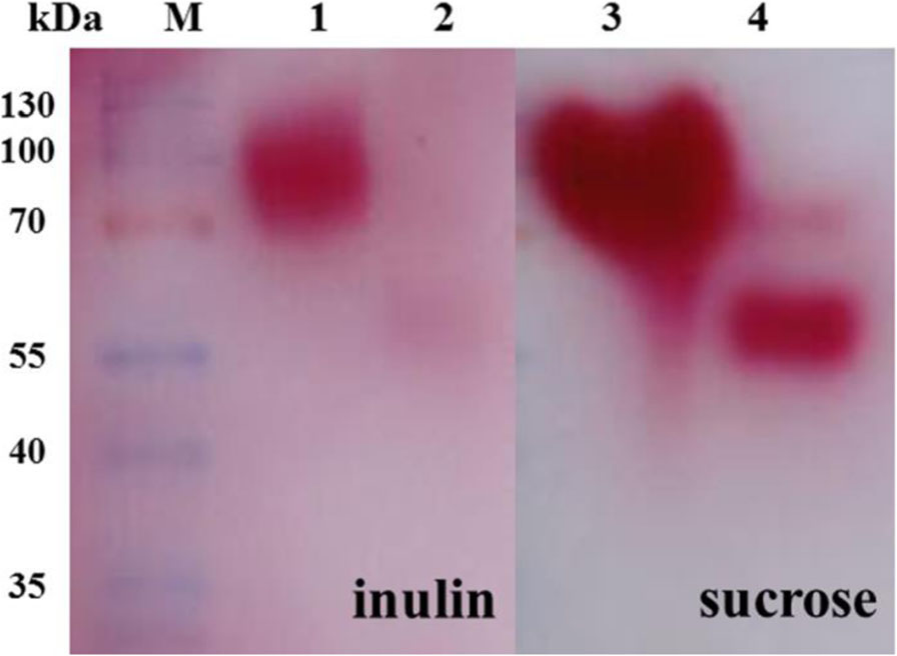
Zymogram analysis of the activity of the glycosylated rKcINU1 and its deglycosylated form drKcINU1. The purified rKcINU1 was digested by Endo F1 to generate the deglycosylated enzyme, designated as drKcINU1, and both were subjected to PAGE analysis, followed by staining with 1% (w/v) 2,3,5-triphenyltetrazolium chloride (TTC), and the enzyme activities were revealed in situ. Lane M: the protein marker, Lane 1: rKcINU1 (with inulin as substrate); Lane 2: drKcINU1 (with inulin as substrate); Lane 3: rKcINU1 (with sucrose as substrate); Lane 4: drKcINU1 (with sucrose as substrate)

In order to investigate the effects of *N*-linked oligosaccharide chains on the hydrolytic activity of rKcINU1, purified rKcINU1 was subjected to Endo F1 treatment under non-denaturing conditions, followed by zymogram analysis and DNS assay to detect enzymatic activity. Endo F1 is not sensitive to protein conformation, and is thus suitable for the cleaving of oligosaccharide chains, even those deeply buried under natural conditions, though it leaves a single GlcNAc unit at the Asn residue. The hydrolytic activity of purified rKcINU1 with glycosylation modification was shown to be higher with both inulin and sucrose as the reaction substrate, compared to that of the deglycosylated rKcINU1 as presented in situ (Fig. 2). In other words, glycosylation modification allowed rKcINU1 to maintain high hydrolytic activity, whereas the removal of oligosaccharide moieties dramatically decreased the enzymatic activity, while still carrying the inner GlcNAc residue. These results confirmed not only the glycosylation modification, but also the fact that the length of oligosaccharide chains contributed to the positive effect on the enzymatic activity.

The DNS method was applied to quantitatively measure the decrement extent of enzyme activity, due to the removal of the *N*-linked glycan chains. After treatment by Endo F1 for 4 h (Additional file 1: Table S2), the hydrolytic activity of rKcINU1 could only keep around 70% against inulin and 63% toward sucrose; and when more glycan chains were removed, the data for both substrates fell to between 30 and 40% when treated for 16 h. These results further strengthened the conclusion that *N*-glycosylation modification was necessary to maintain the enzyme activity of rKcINU1.

### Determination of the glycosylation sites of the wild-type rKcINU1 by mass spectrometry analysis

**Fig. 3:**
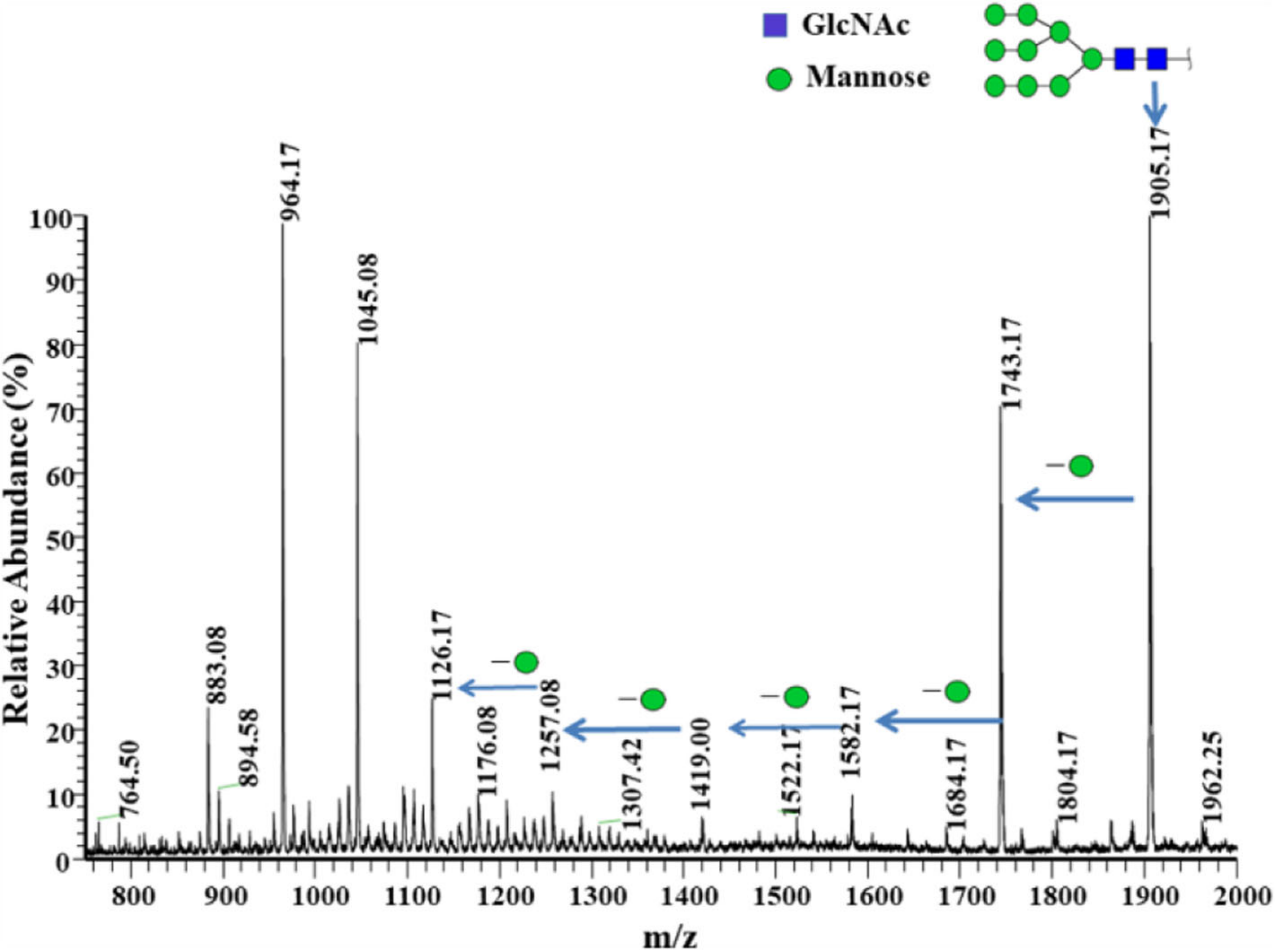
Positive ion ESI-MS analysis of *N*-linked glycan chains for the wild-type rKcINU1. The peak of m/z 1905.17 confirmed the presence of a high mannose oligosaccharide (Man)_7_(GlcNAc)_2_. Series of peaks by 162 Da (the mass of anhydrohexose) demonstrated the glycoform heterogeneity. Solid squares and circles represented *N*-acetylglucosamine (GlcNAc) and Mannose (Man), respectively

In order to identify *N*-linked glycans sites, the purified wild-type rKcINU1 was subjected to trypsin hydrolysis, followed by PNGase F digestion, prior to mass analysis on the LTQ-Orbitrap Elite hybrid mass spectrometer. Mass spectrometry assigned a single deamidation modification to each Asn residue within the Asn-X-Ser/Thr (X ≠ Pro) motif, producing the five sites of Asn-362, Asn-370, Asn-399, Asn-467 and Asn-526 (Additional file 1: Figure S1). While no mass alteration was found on the PNGase F treated trypsin peptides containing Asn-9, Asn-99, Asn-100, Asn-106, Asn-153, Asn-203 or Asn-267, the absence of *N*-glycosylation modification of those putative sites in the wild-type rKcINU1 was demonstrated. Quite interestingly, the authentic glycosylation modification site Asn-399, found in the VFNGSP sequence, was not included in the list of putative sites based on prediction in silicon. The ESI-MS profile from the wild-type rKcINU1 proved the variable high mannose-type oligosaccharides (Man_3–9_GlcNAc_2_) carrying up to nine mannose residues of the *N*-linked glycan chains (Fig. 3). Furthermore, the MALDI-TOF mass spectra of the wild-type rKcINU1 suggested different glycoform ensembles caused by glycoform heterogeneity [20], each with variable numbers of mannose residues, as indicated by arrows for the typical clusters in Fi. 1C. These results demonstrated that only 4 of the 11 predicted *N*-glycosylation sites, plus the Asn-399 site in the wild-type rKcINU1 sequence, were finally modified by the heterogeneous high mannose-type glycan chains.

### Three-dimensional modeling of the wild-type rKcINU1

**Fig. 4:**
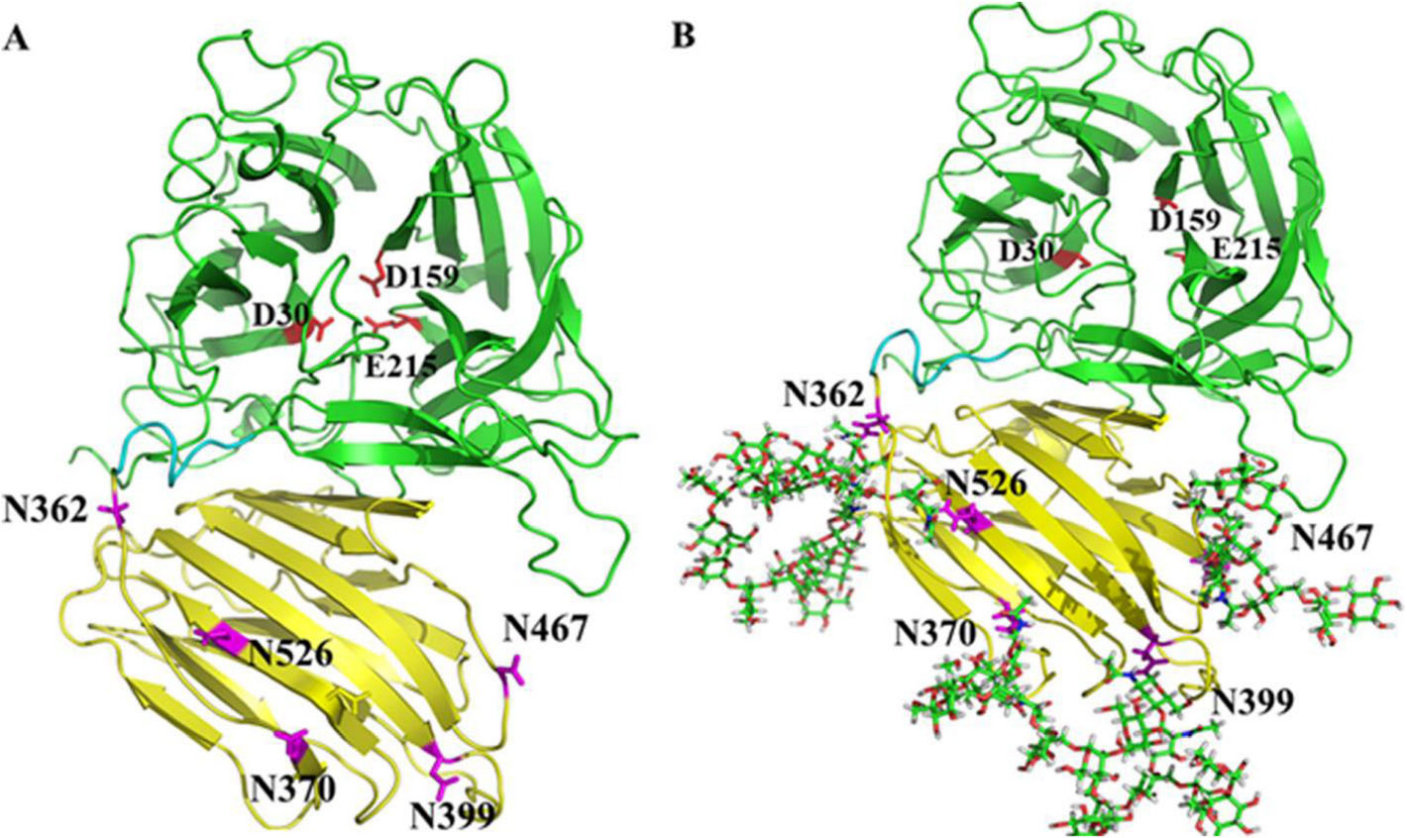
Three-dimensional structure modeling of the wild-type rKcINU1 (**a**) with *N*-glycosylation sites and (**b**) with *N*-linked glycan chains. The protein 3D model was built by the Swiss-Model server, followed by manually docking the (Man)_7_(GlcNAc)_2_ glycan chains to the Asn-362, Asn-370, Asn-399, Asn-467 and Asn-526 residues using the program GLYCAM. The wild-type rKcINU1 folded into two domains, a N-terminal *β*-propeller catalytic domain (in green) and a C-terminal *β*-sandwich domain (in yellow) formed by two antiparallel six-stranded *β*-sheets, and the two domains were linked by a short loop illustrated in cyan. The *N*-glycosylation sites Asn-362, Asn-370, Asn-399, Asn-467 and Asn-526 and *N*-linked glycan chains were indicated by purple sticks and green sticks, respectively, while the catalytic active residues (Asp-30, Asp-159 and Glu-215) were shown as red sticks

Next, the glycoprotein 3D model structure of the wild-type rKcINU1 with (Man)_7_(GlcNAc)_2_ glycans was built (Fig. 4). As illustrated, the wild-type rKcINU1 adopted the bimodular structure typical of the GH32 family. It folded into a five-blade *β*-propeller catalytic domain at the N-terminus. This accommodated for the active site of the enzyme (with each blade composed of four antiparallel *β*-strands with a ‘W’ topology) and a C-terminus *β*-sandwich domain assembled from two six-stranded antiparallel *β*-sheets, with a short loop linking the two domains [4]. Three key acidic amino acid residues were located in the active site, and were responsible for substrate binding and hydrolysis. These included two Asp and one Glu residues in the conserved sequences of GH32 family, namely NDPNG (D acted as anucleophile) and RDP (D acted as a stabilizer of the transient state), as well as EC (E acted as an acid base catalyst).

### The priority of *N*-glycosylation for the C-terminus domain with a unique sequence

**Fig. 5:**
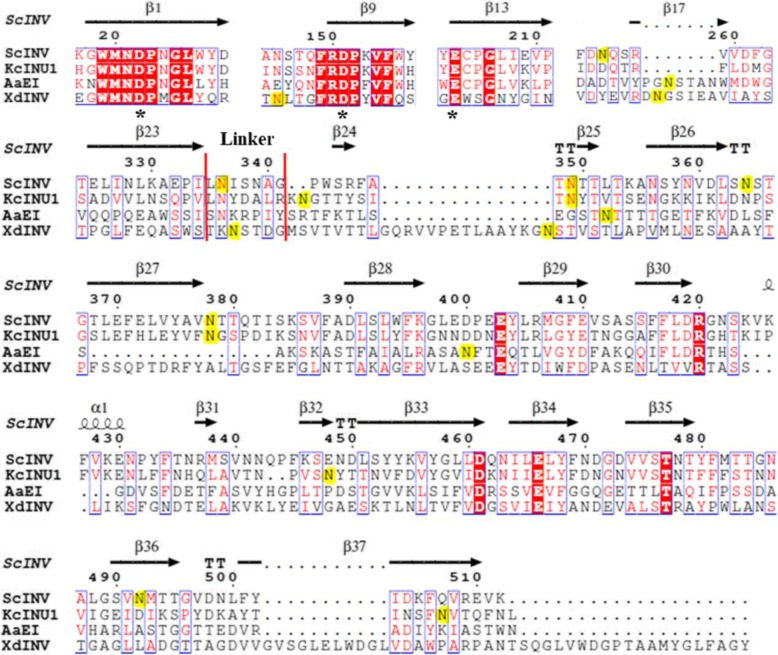
Protein sequence alignment of KcINU1 with three other GH32 members from fungi, including *S.cerevisiae* invertase (ScINV, PDB code 4EQV), *Aspergillus awamorii* exo-inulinase (AaEI, PDB code 1Y9G), and *Xanthophyllomyces dendrorhous β*-fructofuranosidase (XdINV, PDB code 5ANN). Convinced glycosylation sites were highlighted in yellow. The black asterisks indicated residues involved in the catalytic center, and the red squares indicated amino acid similarity as calculated by ESPript. The structural annotation was based on the crystal structure of ScINV

A unique feature of the wild-type rKcINU1 model was that the five glycosylation sites mentioned above were located at the C-terminus *β*-sandwich domain, all of which were placed on the same six-stranded *β*-sheet located opposite the catalytic active center. As shown in the built three-dimensional model (Fig. 4), Asn-362 at the C-terminus domain near the linker was quite close to the entrance for the substrate into the catalytic active cavity, in the upper open pocket of the ‘W’ topology. Meanwhile, the other *N*-glycosylation sites were located on the *β*-strands, or turns in the C-terminus *β*-sandwich domain at the reverse side of the catalytic cavity, isolated from the catalytic active pocket. The multiple sequence alignment of KcINU1 (native sequence), along with the other three members of the family GH32 whose glycosylation sites have been identified in previous research from fungi, was used (Fig. 5). However, no conservative *N*-glycosylation sites were found in any of the 4 enzymes, even between KcINU1 and ScINV, which shared the highest sequence homogeneity of up to 52%, with only two *N*-glycosylation sites conserved: Asn-370 and Asn-399 in KcINU1 equivalent to Asn-349 and Asn-378 in ScINV, respectively. Meanwhile, Asn-362, Asn-467 and Asn-526 in KcINU1 represent “unique” *N*-glycosylation sites, which are absent in all other enzymes analyzed. The most remarkable feature was that no authentic *N*-glycosylation modification in KcINU1 was located in the N-terminal catalytic domain, despite its seven putative glycosylation sites in this sequence observed from the prediction results (Fig. 1 b). This is unlike three other enzymes possessing *N*-linked glycan sites in the catalytic domain and the *β*-sandwich domain simultaneously. All of these results showed that the C-terminal sequence of the KcINU1 may be unique among the GH32 family in fungi, thus ensuring the priority of glycosylation modification in the C-terminus *β*-sandwich domain, instead of the N-terminal catalytic domain. Based on previous research, an Asn residue in an Asn-X-Thr sequon was more readily glycosylated than that in an Asn-X-Ser sequon [38]. In addition, the middle residue with small hydrophobic and positively charged side chain of an Asn-X-Ser consensus motif was more efficiently *N*-glycosylated [39]. Recently, Huang et al. discovered the enhanced aromatic sequon (Phe-X-Asn-X-Thr and Phe-X-X-Asn-X-Thr) could be efficiently *N*-glycosylated, namely aromatic residues, especially Trp and sulfur-containing residues at the X_1_ position in five-residue sequon X_1_-X_2_-N-X_3_-S/T, improved *N*-glycosylation efficiency, while positively charged residues such as Arg suppressed *N*-glycosylation [40]. However, none of the theories described above could rationally explain the high efficiency of glycosylation at Asn-399 within the VFNXSP sequence. As shown in Fig. 1 b, Asn-399 actually possessed little likelihood of being glycosylated according to the prediction result compared to Asn-9, Asn-99, Asn-203 and Asn-267 (all in NXT motifs), which in the end were not glycosylated for unknown reasons. Interestingly, Asn-399 in the VFNXSP sequence was located right between two consecutive NXT motifs (NXT-NXT-NXS-NXT-NXT), whose binding to oligosaccharyltransferase (OST) may hinder the potential skipping of OST through Asn-399, also for unknown reasons [39].

In any case, the unique sequence above was favorable to *N*-glycosylation modification, and thus provided a novel insight of artificially designing the glycosylation modification in the C-terminal *β*-sandwich domain to fine-tune the enzyme function. Furthermore, in previous research regarding the bimodular CBH enzymes, it was the N-terminus domain incorporating the catalytic cavity that was more ready to be *N*-glycosylated, while the C-terminal domain and the linker between the two were sensitive to *O*-glycosylation [21, 22, 41, 42]. However, in this case it was observed that all five *N*-glycosylation modification sites were located in the C-terminus non-catalytic domain, far from the active cavity, while still efficiently regulating the enzymatic activity. This implied a novel regulation mechanism for the enzyme properties, as well as a model of bimodular enzymes, by which to study the effects of *N*-glycosylation in non-catalytic domains on enzyme properties.

### Mutagenesis and expression of rKcINU1

**Fig. 6:**
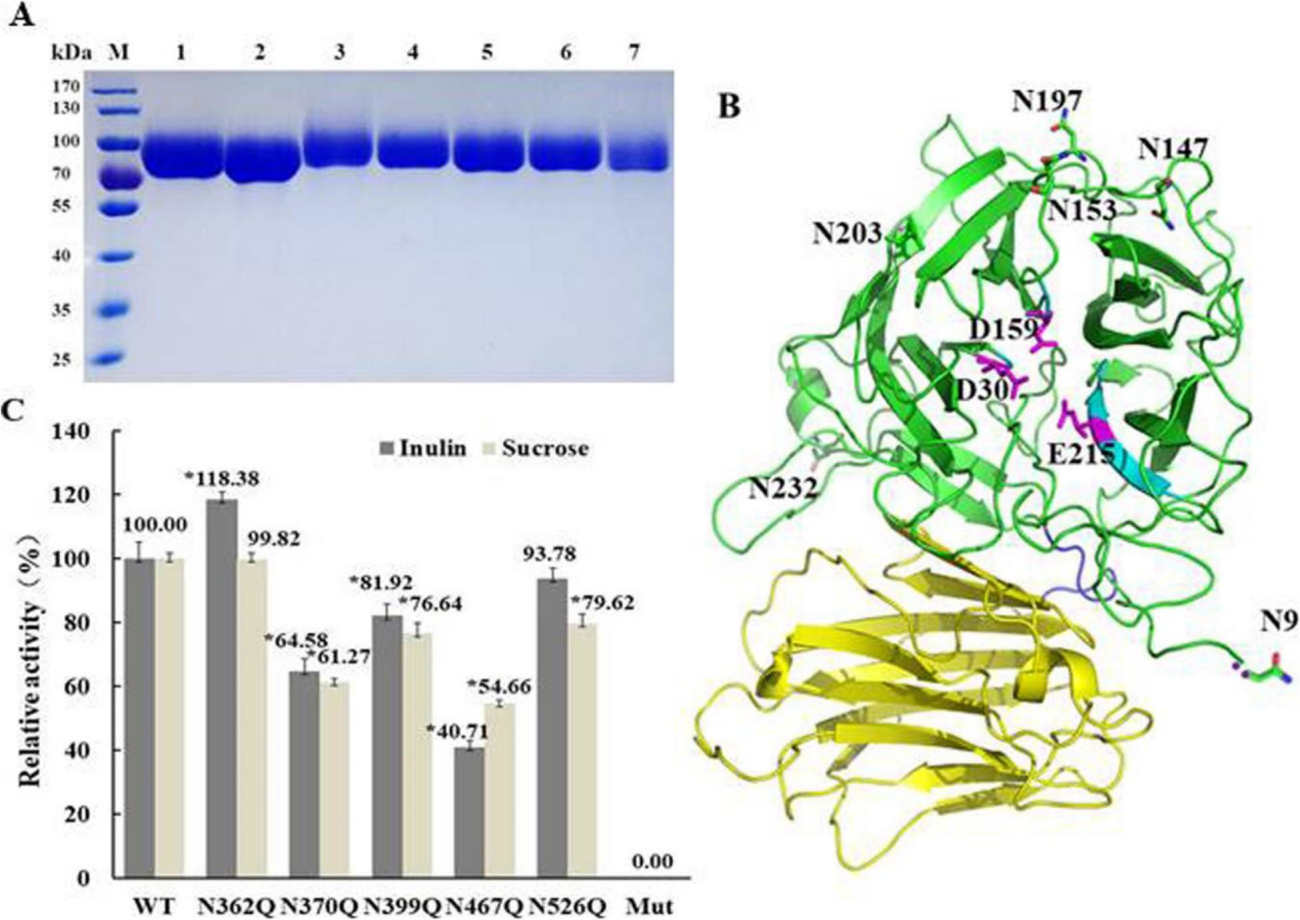
Purification, glycan sites and specific enzyme activity analysis of the mutants with altered *N*-glycosylation modification. **a**, SDS-PAGE analysis of the purified wild-type rKcINU1 and its mutants. Lane M: the protein marker; Lane 1: the purified rKcINU1; Lane 2: the purified N362Q; Lane 3: the purified N370Q; Lane 4: the purified N399Q; Lane 5: the purified N467Q; Lane 6: the purified N526Q; Lane 7: the purified Mut. **b**, the 3D model of Mut with six novel *N*-glycosylation sites completely different from the wild-type. The six new glycosylation sites (Asn-9, Asn-147, Asn-153, Asn-197, Asn-203, Asn-232) as indicated by sticks were all located at the N-terminal catalytic region instead of the C-terminal domain. Its catalytic active sites (Asp-30, Asp-159 and Glu-215) were indicated by sticks in purple. **c**, the relative specific enzyme activity of mutants. The value given represented the average of three replications, and the specific activity of the wild-type was designated as 100%. * *P* < 0.01

To elucidate the precise role which *N*-glycosylation modification played in maintaining the activity and stability of rKcINU1, each glycosylation site was mutated to Gln by means of site-directed mutagenesis. In addition, a mutant without any *N*-glycosylation sites (*mut*) was obtained by mutating all five Asn residues mentioned above to Gln. The DNA sequencing of the entire gene fragment confirmed that the amino acid substitution and no other undesired mutations. Then, the six mutated genes were successfully expressed in *P. pastoris*X-33 and purified by nickel affinity chromatography, yielding six recombinant proteins, i.e. N362Q, N370Q, N399Q, N467Q, N526Q and Mut (Fig. 6 a). Among these the variant Mut was expected to be expressed without *N*-glycosylation. However, to some extent *N*-glycosylation was still unexpectedly detected in the Mut from the smear band between 70 and 100 kDa in SDS-PAGE as the wild-type. In order to determine the exact glycosylation sites, the Mut was hydrolyzed by trypsin, followed by PNGase F treatment, then applied to the LTQ-Orbitrap Elite hybrid mass spectrometer. Astonishingly, mass spectrometry revealed six new *N*-linked glycan sites which were all located at the N-terminal catalytic domain (Fig. 6 b), i.e. Asn-9, Asn-147, Asn-153, Asn-197, Asn-203, Asn-232 (Additional file 1: Figure S2). This once again clearly illustrated the priority of *N*-glycosylation modification at the C-terminal sequence.

In order to elucidate the glycan pattern, purified proteins with single-site mutation digested with PNGase F were analyzed via mass spectrometry. The ESI-MS profiles revealed that the *N*-linked glycans of mutants N362Q, N370Q, N399Q, N467Q and N526Q respectively represented Man_4–9_GlcNAc_2_, Man_4, 6–9_GlcNAc_2_, Man_4, 6–9_GlcNAc_2_, Man_4, 7–9_GlcNAc_2_, and Man_4, 7–9_GlcNAc_2_ (Additional file 1: Figure S3).

### *N*-linked Glycans are crucial for enzyme activity

The specific activities of the wild-type rKcINU1 and its mutants against inulin and sucrose are shown in Fig. 6 c. In general, mutants with a single *N*-glycosylation site mutated (N362Q, N370Q, N399Q, N467Q and N526Q) kept activity to some extent toward inulin and sucrose, whereas the Mut lost all hydrolytic activity, likely due to defective protein folding caused by incorrect glycosylation. This fact suggested the conclusion that the *N*-glycosylation modification at the C-terminal *β*-sandwich domain was necessary for the correct folding of rKcINU1, and essential for keeping an active form. Among the single mutants, N362Q led to an 18% increase in the specific activity against inulin in comparison with the wild-type, without any effect of hydrolysis activity toward sucrose on the other hand. This demonstrated that the *N*-glycosylationat Asn-362 negatively affected the rKcINU1 activity. On the contrary, other single mutations, such as N370Q, N399Q, N467Q and N526Q, resulted in a decrease of the specific activity to various degrees toward inulin and sucrose compared to the wild-type. Among these, the most dramatic decline of specific activity was observed in the case of N467Q, respectively to 40.71 and 54.66% of enzyme activity against inulin and sucrose compared to the wild-type, thus indicating that the glycosylation at Asn-467 played the most essential role in sustaining the enzymatic activity of the wild-type. In addition, N526Q presented the least significant reduction in specific activity, both for inulin and sucrose as a substrate. These results implied the potential regulation of *N*-glycosylation in maintaining enzymatic activity with contribution to various extents, according to the glycosylated site. Meanwhile, the glycosylation at Asn-467 exhibited the most outstanding effect on maintaining enzyme activity, while the glycosylation at Asn-362 led to a negative effect in the hydrolysis activity of the enzyme toward inulin.

Next, the kinetic properties with sucrose as the substrate for both the wild-type rKcINU1 and its mutants were investigated (Table 1). The data showed that the *K*_m_ values of single-site mutants were higher than that of the wild-type. They also had lower apparent catalytic efficiency (*k*_cat_/*K*_*m*_), such as N467Q with the lowest activity among all single-site mutants, presenting a 46-fold lower *k*_cat_/*K*_*m*_ value and 2-fold higher *K*_*m*_ value compared to the wild-type. These results indicated that *N*-glycosylation modification could positively affect the substrate-enzyme affinity, which may be a key reason for the decline in the activities of the mutants.

**Table 1:** Kinetics parameters of the wild-type rKcINU1 and its mutants

Sample	*K*_*m*_ (μM)	*V*_*max*_ (μM•min^−1^•mg^− 1^)	*k*_*cat*_ (s^− 1^)	*k*_*cat*_*/K*_*m*_ (μM^− 1^•s^− 1^)
WT	32.05 ± 1.53	3240.35 ± 77.93 ^a^	3269.51	102.01
N362Q	33.04 ± 4.11	3274.40 ± 214.24	3303.87	100.00
N370Q	32.73 ± 4.99	1718.90 ± 143.78	1734.37	52.99
N399Q	24.89 ± 1.07	2338.92 ± 50.92	2359.97	94.82
N467Q	76.29 ± 16.21	167.10 ± 25.47	168.60	2.21
N526Q	37.97 ± 3.4	3023.79 ± 163.24	3051.00	80.35

The value represented an average of three replications. ^a^mean ± SD

### Effect of *N*-glycosylation on the second structure of rKcINU1

**Fig. 7:**
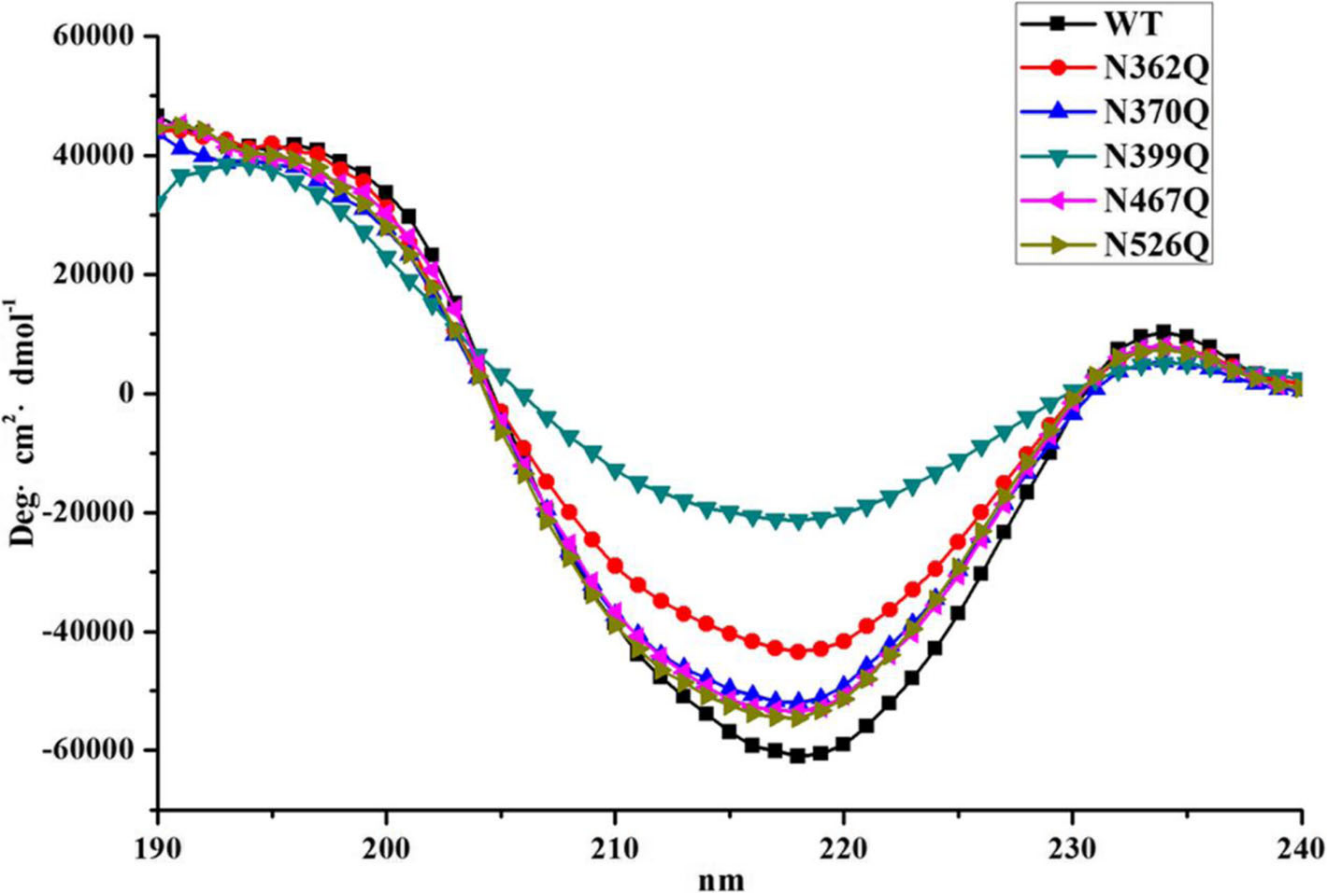
Far-UV CD spetra of the wild-type rKcINU1 and its mutants. The purified samples were measured at a protein concentration of 0.2 mg/mL in 20 mM phosphoric acid (pH 4.5). As illustrated, the strong negative peaks around 218 nm in the far-UV CD spectra implied the typical feature of the enzymatic secondary structure rich in *β*-sheet. The following quantitative analysis with CDpro software proved the altered ratios of secondary structure elements among the mutants and the wild-type

To elucidate whether *N*-glycosylation modification had an effect on the protein conformation, the secondary structures of the wild-type rKcINU1 and its mutants were determined using CD spectra in the far-UV spectrum region (Fig. 7, Table 2). The far-UV CD spectrum of the wild-type exhibited a strong negative peak around 218 nm, a typical feature for the protein rich in *β*-sheet [43], further followed by a quantitative analysis giving the ration of *β*-sheet of 42.11%, which is consistent with the feature of the GH32 family with abundant *β*-sheet. The CD spectra of N362Q, N370Q, N467Q and N526Q showed similar profiles to that of the wild-type, with an increase in the negative peak approximately at 218 nm. This corresponded to an increase in content of both the *β*-sheet and random coil conformation, but a decline in the ratio of *α*-helix. For N399Q, an additional positive peak around 192 nm was observed, except for the regular negative peak around 218 nm, which was reflected with the highest increase (by 7%) in the ratio of *β*-helix from 42.11 to 44.98%, as well as a decrease in random coil (by 5%) and *β*-turn (by 6%). These results suggested that glycosylation mutations altered the secondary structure of the rKcINU1. Therefore, it was assumed that the change in the secondary structure may have led to altered enzyme activity through fine-tuning substrate affinity.

**Table 2:** The secondary structure proportion of the wide-type rKcINU1and its mutants

Sample	*α*-helix (%)	*β*-sheet (%)	*β*-turn (%)	Random coil (%)
WT	6.23	42.11	21.40	30.27
N362Q	4.27	43.79	21.55	30.41
N370Q	5.34	42.35	21.66	30.65
N399Q	6.26	44.98	20.16	28.62
N467Q	5.52	42.53	21.53	30.44
N526Q	5.66	42.27	21.58	30.49

### Effect of *N*-glycosylation on thermal stability of rKcINU1

Glycosylation is closely related to thermal stability in glycoproteins. Therefore, the impact of *N*-glycosylation on the thermostability of rKcINU1 was evaluated by DSC, and the results are presented in Table 3. In general, the melting temperature values (*T*_*m*_) of single-site glycosylation mutants were lower than that of the wild-type (88.89 °C), whereas N362Q outweighed the other mutants with 2.91 °C compared with the wild-type. In other words, the protein with removal of the *N*-linked glycan chains at Asn-362 would lead to significant decrease in thermostability, thereby implying that the glycosylation at Asn-362 contributed to stabilizing the structure of rKcINU1.

**Table 3:** Thermodynamics data of the wild-type rKcINU1 and its glycosylated mutants

Sample	*T*_*m*_ (°C)	*ΔH* (kJ/mol)	*ΔS* (kJ/mol·K)
WT	88.89	272.6	0.7184
N362Q	85.98	261.9	0.7629
N370Q	86.11	258.0	0.6646
N399Q	87.74	248.2	0.6728
N467Q	88.78	267.9	0.7676
N526Q	89.66	277.1	0.7205

In addition, only the *T*_*m*_ of N526Q was slightly increased, as shown in Table 3, partially due to its location too close to glycosylation site Asn-362. This might owe to the intensive distribution of glycan chains, which contributed to the interactions between glycans instead of the protein and glycan, which is not conducive to protein thermostability. In contrast, more dispersed glycosylation sites would favor protein-glycan interactions, thus enhancing thermostability [20].

Furthermore, it was concluded that the glycan position, rather than the glycan pattern, had important effects on enzyme thermostability [20], just as the ESI-MS analysis (Additional file 1: Figure S3) indicated that the glycan patterns of N370Q were similar to those of N399Q, and the glycan patterns of mutant N467Q were consistent with those of N526Q. However, each mutant exhibited varying thermostability.

### Mechanism of the effect of *N*-glycosylation on enzyme properties

From all the data above, it was concluded that the *N*-glycosylation at C-terminus *β*-sandwich domain had the following functions. First of all, the *N*-linked glycans could stabilize the C-terminal domain by keeping the *β*-strands in appropriate conformation, which was responsive to both enzymatic activity and thermostability. For example, Asn-467 was located at the loop region between two antiparallel *β*-sheets in the C-terminal domain. It was concluded that the glycan chains at this site were involved in stabilizing the conformation of the *β*-sandwich domain, similarly to those at Asn-297 of the immunoglobulin G1 Fc, which played a role in stabilizing the C’E loop through intramolecular interactions between carbohydrate and amino acid residues. Without these the Cγ2 domain would collapse, further rendering Fc incapable of binding FcγRІІІA [44, 45]. Another case was the *N*-glycosylation of the ectodomain (ECD) in the epidermal growth factor receptor (EGFR), which was crucial for the conformational arrangement of the ECD thus effecting its orientation to the membrane and the ligand-binding function of EGFR [46].

In this research, the *N*-glycan chains at Asn-467 likely kept the two antiparallel *β*-sheets positioned in the relative stable orientation. The *N*-linked glycan chains could stringently limit the motion of the amide nitrogen of the glycosylation site, as well as rigidifying the residues proximal to the glycosylation site, thereby stabilizing the region surrounding the glycosylation site with increased overall protein stability simultaneously [47]. At the same time, the *N*-linked glycans could also induce the formation of the secondary structure of the nascent polypeptide proximal to the glycosylation site, thereby enabling the protein to be folded into the desired conformation [48–50]. When this glycosylation site was removed via mutagenesis, the two antiparallel *β*-sheets topology may collapse due to the loss of the support of glycan chains, thereby leading to a dramatic decrement of enzyme activity, as proven by the 2-fold higher *K*_*m*_ and 46-fold lower *k*_*cat*_/*K*_*m*_.

Another function of *N*-linked glycans at the site close to the linker between the catalytic domain and *β*-sandwich domain could enhance the overall protein stability, albeit with deterioration in enzymatic activity caused by steric hindrance to adjacent *N*-glycans. For Asn-362, the glycosylation site was located adjacent to the linker between the catalytic domain and *β*-sandwich domain, which may maintain the relative conformation of the two domains mentioned above via increased rigidity of peptides nearby. Meanwhile, the glycosylation performed as a steric hindrance to the adjacent *N*-glycans whose interaction with the substrate was interfered with, thereby leading to delayed access of the substrate into the catalytic center. This in turn negatively regulated the enzyme activity, especially for high polymerization substrates like inulin, but without efficiently affecting the activity with low-molecular-weight substrates like sucrose.

In general, all five glycosylation sites were located in the *β*-sandwich domain, away from the catalytic active center with Asp-30, Asp-159 and Glu-215 at the bottom of the active pocket, thus making it difficult for the glycan chains to gain access to the core amino acid residues in the active pocket, where they could produce direct interaction. Consequently, the regulation of enzyme activity by the *N*-linked glycan chains originated from the stabilization of the configuration of the *β*-sandwich domain and the ease of capturing or transferring the substrate to the active center, which altered the micro-environment closely around the catalytic pocket with higher substrate concentration. This is quite similar to the functions of CBM, i.e. the *N*-linked glycan chains endowed the *β*-sandwich domain with the functions of CBM. This discovery provided a novel aspect by which to fine-tune the enzyme with desired characters for industrial enzymes.

## Conclusions

In this study, the effects of glycosylation on the rKcINU1 activity, secondary structure and thermal stability was studied, uncovering a unique feature of the enzyme rKcINU1 that five glycosylation sites were all located at the C-terminal *β*-sandwich domain, away from the catalytic domain, which could still efficiently regulate the enzyme activity by stabilizing the configuration of the *β*-sandwich region and favoring the substrate into the catalytic center, which increased the substrate concentration in the micro-environment around the active pocket. Therefore it was believed the *N*-linked glycan chains in the non-catalytic domain endowed the C-terminal *β*-sandwich domain with the functions of CBM. This discovery provided a new manipulation strategy for glycoengineering the inulinase from fungi as well as other industrial enzymes from GH32 family.

## Supplementary information

**Additional file 1: **Materials and Methods about Site-Directed Mutagenesis, Protein Expression and Purification, Glycosidase Treatment, Zymogram Analysis, Enzymes Activity Assays and Mass Spectrometry Analysis. **Figure S1.** LC-MS/MS spectrum analysis of the glycosylation sites within the wild-type rKcINU1. The sample was treated by trypsin followed with PNGase F digestion and then subjected to LC-MS/MS. The peak with m/z of 976.95, 694.66, 942.49, 1188.59 and 817.90 confirmed the presence of deamidation modification on (A) Asn-362, (B) Asn-370, (C) Asn-399, (D) Asn-467 and (E) Asn-526, respectively (substitution of Asn with Glu residue). Each peptide sequence was shown in the panel and the arrows indicated the *N*-glycosylation sites. **Figure S2.** LC-MS/MS spectrum analysis of the glycosylation sites within the variant Mut. The sample was treated by trypsin followed with PNGase F digestion and then subjected to LC-MS/MS. The peak with m/z of 621.32, 1298.60, 1298.60, 990.45, 990.45 and 874.37 confirmed the presence of deamidation modification on (A) Asn-9, (B) Asn-147, Asn-153 (C) Asn-197, Asn-203 and (D) Asn-233, respectively (substitution of Asn with Glu residue). Each peptide sequence was shown in the panel and the arrows indicated the *N*-glycosylation sites. **Figure S3.** Positive ion ESI-MS analysis of *N*-linked glycan chains for the single-sited mutants of (A) N362Q, (B) N370Q, (C) N399Q, (D) N467Q and (E) N526Q, respectively. The peak of m/z 1905.17 confirmed the presence of the high mannose oligosaccharide (Man)_7_(GlcNAc)_2_. Series of peaks by 162 Da (the mass of anhydrohexose) demonstrated the glycoform heterogeneity. Solid squares and circles represented *N*-acetylglucosamine (GlcNAc) and Mannose (Man), respectively. **Table S1.** Primers of the site-directed mutagenesis. **Table S2.** The enzyme activity of the wildtype rKcINU1 treated with Endo F1

## Data Availability

We provide all the necessary data for the publication of this article. All additional data is present in the additional material.

## Abbreviations

BMGY: Buffered Glycerol-complex Medium
BMMY: Buffered Methanol-complex Medium
YNB: Yeast Nitrogen Base
YPDSZ: Yeast Extract Peptone Dextrose Medium with Sorbitol
