# Supplementary material for: Unique N-glycosylation of a recombinant exo-inulinase from Kluyveromyces cicerisporus and its effect on enzymatic activity and thermostability
Source: J Biol Eng. 2019 Oct 29;13:81. doi: 10.1186/s13036-019-0215-y (PMC6844067; doi:10.1186/s13036-019-0215-y)
Supplement: Supplementary file 1 — Additional file 1: Materials and Methods about Site-Directed Mutagenesis, Protein Expression and Purification, Glycosidase Treatment, Zymogram Analysis, Enzymes Activity Assays and Mass Spectrometry Analysis. Figure S1. LC-MS/MS spectrum analysis of the glycosylation sites within the wild-type rKcINU1. The sample was treated by trypsin followed with PNGase F digestion and then subjected to LC-MS/MS. The peak with m/z of 976.95, 694.66, 942.49, 1188.59 and 817.90 confirmed the presence of deamidation modification on (A) Asn-362, (B) Asn-370, (C) Asn-399, (D) Asn-467 and (E) Asn-526, respectively (substitution of Asn with Glu residue). Each peptide sequence was shown in the panel and the arrows indicated the N-glycosylation sites. Figure S2. LC-MS/MS spectrum analysis of the glycosylation sites within the variant Mut. The sample was treated by trypsin followed with PNGase F digestion and then subjected to LC-MS/MS. The peak with m/z of 621.32, 1298.60, 1298.60, 990.45, 990.45 and 874.37 confirmed the presence of deamidation modification on (A) Asn-9, (B) Asn-147, Asn-153 (C) Asn-197, Asn-203 and (D) Asn-233, respectively (substitution of Asn with Glu residue). Each peptide sequence was shown in the panel and the arrows indicated the N-glycosylation sites. Figure S3. Positive ion ESI-MS analysis of N-linked glycan chains for the single-sited mutants of (A) N362Q, (B) N370Q, (C) N399Q, (D) N467Q and (E) N526Q, respectively. The peak of m/z 1905.17 confirmed the presence of the high mannose oligosaccharide (Man)7(GlcNAc)2. Series of peaks by 162 Da (the mass of anhydrohexose) demonstrated the glycoform heterogeneity. Solid squares and circles represented N-acetylglucosamine (GlcNAc) and Mannose (Man), respectively. Table S1. Primers of the site-directed mutagenesis. Table S2. The enzyme activity of the wildtype rKcINU1 treated with Endo F1 [file 13036_2019_215_MOESM1_ESM.docx]

**Additional file 1**

**Unique *N*-Glycosylation of a Recombinant Exo-inulinase from *Kluyveromyces cicerisporus* and Its Effect on Enzymatic Activity and Thermostability**

Junyan Ma^1, 2†^, Qian Li^2†^, Haidong Tan^1^, Hao Jiang^3^, Kuikui Li^1^,

Lihua Zhang^3^, Quan Shi^3^, Heng Yin^1*^

^1^Natural Products and Glyco-Biotechnology Research Group, Liaoning Province Key Laboratory of Carbohydrates, Dalian Institute of Chemical Physics, Chinese Academy of Sciences, Dalian 116023, China

^2^Liaoning Province Key Laboratory of Bio-Organic Chemistry, Dalian University, Dalian 116622, China

^3^Dalian Institute of Chemical Physics, Chinese Academy of Sciences, Dalian 116023, China

^†^Jun-Yan Ma and Qian Li contributed equally to this work

*Correspondence: yinheng@dicp.ac.cn

**Additional Materials and Methods**

**Site-Directed Mutagenesis, Protein Expression and Purification**

The recombinant expression vectors carrying wild-type or mutated genes were linearized with *Sac*I and then transformed into *P. pastoris* X-33 by electroporation. Positive transformants were selected on YPDSZ medium and confirmed by PCR amplification analysis. The recombinant strains containing the target genes were used for expression as described elsewhere [[35](#_ENREF_35)]. And then, the harvested culture supernatant was purified using Ni-NTA sepharose^TM^ excel chromatography column (GE Healthcare, Amersham, Sweden) according to the procedure published previously [35]. The purity of active elution fractions was confirmed by sodium dodecylsulfate poly acrylamide gel electrophoresis (SDS-PAGE) using Coomassie Blue R-250 staining. The eluted fraction containing the wild-type rKcINU1 or its mutants were concentrated and processed with buffer exchange by an Amicon Ultra 10-kDa molecular weight cut-off (Millipore Corporation, Billerica, MA) for the structure and function analysis. Protein concentration was determined with the BCA Protein Assay Kit (Beyotime Biotechnology, Beijing, China) using bovine serum albumin as a standard.

**Glycosidase Treatment****, Zymogram Analysis and Enzymes Activity Assays**

The deglycosylation reaction of the purified wild-type rKcINU1 using PNGase F was carried out under denaturing and non-denaturing conditions. Briefly, The purified enzyme (3 μg) was boiled for 10 minutes in denaturing buffer containing 0.5% (w/v) SDS and 40 mM DTT, and then incubated with PNGase F at 37°C for 2 h in 50 mM sodium phosphate buffer (pH 7.5) supplemented with 1% (w/v) NP-40. As for the non-denaturing condition, 3 µg of purified protein was directly incubated with PNGase F in 50 mM sodium phosphate buffer (pH 7.5) at 37°C for 2 h without boiling, followed with SDS-PAGE analysis.

To obtain an active form of deglycosylated rKcINU1 (designated as drKcINU1), the purified rKcINU1 (112.7 μg) was also treated with 0.3 units of Endo F1 under non-denaturing condition in 20 mM Tris-HCl buffer (pH 7.5) at 37°C for 4 h. Meanwhile, the same amount of purified rKcINU1 without Endo F1 was processed under identical conditions as control. At the end of the reactions, samples were subjected to zymogram analysis and enzyme activity assays. For zymogram analysis, the 10% non-denaturing polyacrylamide gel electrophoresis was performed, followed by hydrolytic activity detection in situ via incubating the gels in 2% (w/v) inulin (Sigma, Saint Louis, MO, USA), and then stained with 1% (w/v) 2,3,5-triphenyltetrazolium chloride in 0.25 M NaOH as described previously [5, 35]. Sucrose (2%, w/v) (Sigma, Saint Louis, MO, USA) was also used as a substrate in the zymogram analysis.

The hydrolytic activity of wild-type rKcINU1 and drKcINU1 as well as its variants were determined by the dinitrosalicylic acid (DNS) colorimetric method as referred previously [35]. Four micrograms of each purified enzyme mentioned above was incubated with 2% (w/v) inulin from dahlia tubers in 100 mM acetate buffer (pH 4.5) for 10 min at 55°C. Each enzyme was inactivated by boiling for 10 min and the amount of reducing sugar was measured by DNS method. For the substrate of sucrose, analysis was conducted similarly just with 0.4 μg of each purified enzyme. One unit (U) of enzyme activity is defined as the amount of enzyme required to generate 1 µmol of reducing sugar in one minute under each assay condition.

**Mass Spectrometry Analysis**

The purified wild-type rKcINU1 and Mut were diluted by 20 mM ammonium bicarbonate (pH 8.0) to 0.75 mg/mL and denatured by heating at 95°C for 5 min. After cooling, they were reduced in 10 mM dithiothreitol at 56°C for 1.5 h, followed by cysteine residues alkylation in 20 mM iodoacetamide at room temperature for 40 min in the dark. Subsequently, protein hydrolysis was performed by adding trypsin into the aforementioned solution for 24 h at 37°C. Trifluoroacetic acid was added to terminate the reaction. The peptides after trypsin digestion were desalted by a homemade reversed-phase C18 column and further lyophilized by a vacuum concentrator (Thermo Fisher, San Jose, CA, USA). The enrichment of glycopeptides was performed using commercial hydrazine-functionalized agarose gel (Affi-gel Hz, Bio-Rad, Shanghai, China). In brief, the lyophilized peptides were resolubilized in 100 mM pH 5.5 acetic acid buffer containing 150 mM sodium chloride, and reacted with 100 mM sodium periodate for 1 h in the dark. Then, they were reduced in 100 mM sodium sulfite, followed by incubation with the hydrazine-functionalized agarose gel beads with the volume ratio of 3:1 at room temperature for overnight. After incubation, the microspheres were eluted in turn with 1.5 M NaCl, methanol, water, acetonitrile and 20 mM ammonium bicarbonate buffer (pH 8.0, ^18^O). The eluted fractions were treated with PNGase F at 37°C for 24 h and then centrifuged to obtain the desired peptides.

The peptides were subjected to mass spectrometry analysis on a nano-RPLC-ESI-MS/MS system with a LTQ Orbitrap Velos mass spectrometer, Accela 600 pump and Accela autosampler (Thermo Fisher, San Jose, CA, USA) as described by Jiang [36]. The spectra data were processed using the Mascot Server (version 2.3.2) with tolerance parameters set at 10 ppm for parent ions and 0.05 Da for fragment ions, respectively. Trypsin cleavage constraint was applied in searching of peptides, and a maximum of two missed cleavage was allowed. The selection variable modifications included carbamidomethyl of Cys residues, oxidation of Met residues, acetyl of protein N-terminal and deamidation of Asn residues. PNGase F cleaved the glycosidic bond between the Asn and the linked glycans, yielding a conversion from Asn to Asp at the same time, resulting in a monoisotopic mass shift of +2.98 Da in O^18^ buffer. Mass spectrum data were then screened in the database incorporation of both the wild-type rKcINU1 and the Mut sequences. *N*-glycosylation sites can only be confirmed under both conditions: (1) the glycopeptides have the deamidation modification at Asn residues; (2) the peptides contain the Asn-X-Ser or Asn-X-Thr sequon (where X cannot be Pro).

**Fig. S1.** LC-MS/MS spectrum analysis of the glycosylation sites within the wild-type rKcINU1. The sample was treated by trypsin followed with PNGase F digestion and then subjected to LC-MS/MS. The peak with m/z of 976.95, 694.66, 942.49, 1188.59 and 817.90 confirmed the presence of deamidation modification on (A) Asn-362, (B) Asn-370, (C) Asn-399, (D) Asn-467 and (E) Asn-526, respectively (substitution of Asn with Glu residue). Each peptide sequence was shown in the panel and the arrows indicated the *N*-glycosylation sites.

**
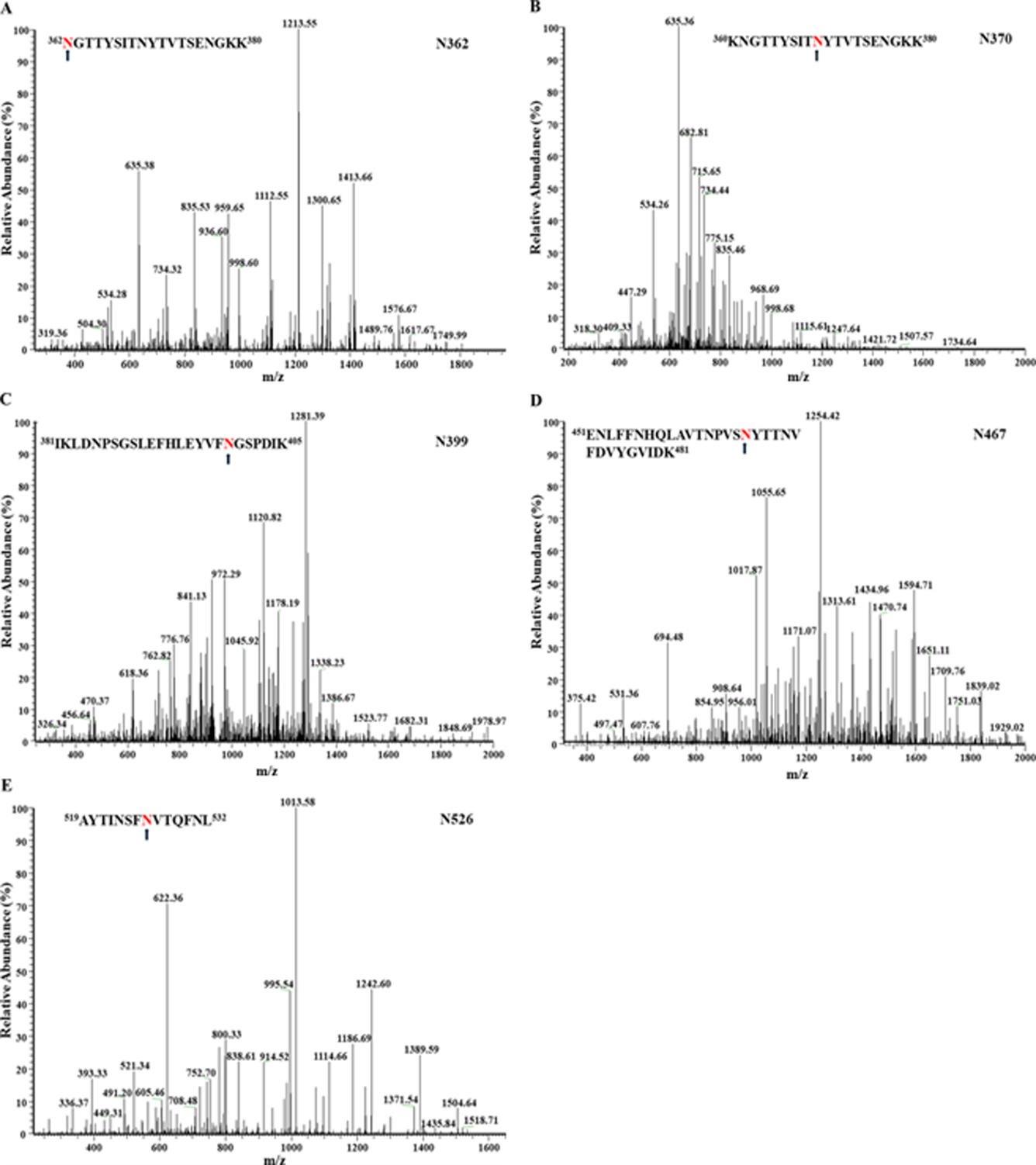
**

**Fig. S2.** LC-MS/MS spectrum analysis of the glycosylation sites within the variant Mut. The sample was treated by trypsin followed with PNGase F digestion and then subjected to LC-MS/MS. The peak with m/z of 621.32, 1298.60, 1298.60, 990.45, 990.45 and 874.37

confirmed the presence of deamidation modification on (A) Asn-9, (B) Asn-147, Asn-153 (C) Asn-197, Asn-203 and (D) Asn-233, respectively (substitution of Asn with Glu residue). Each peptide sequence was shown in the panel and the arrows indicated the *N*-glycosylation sites.


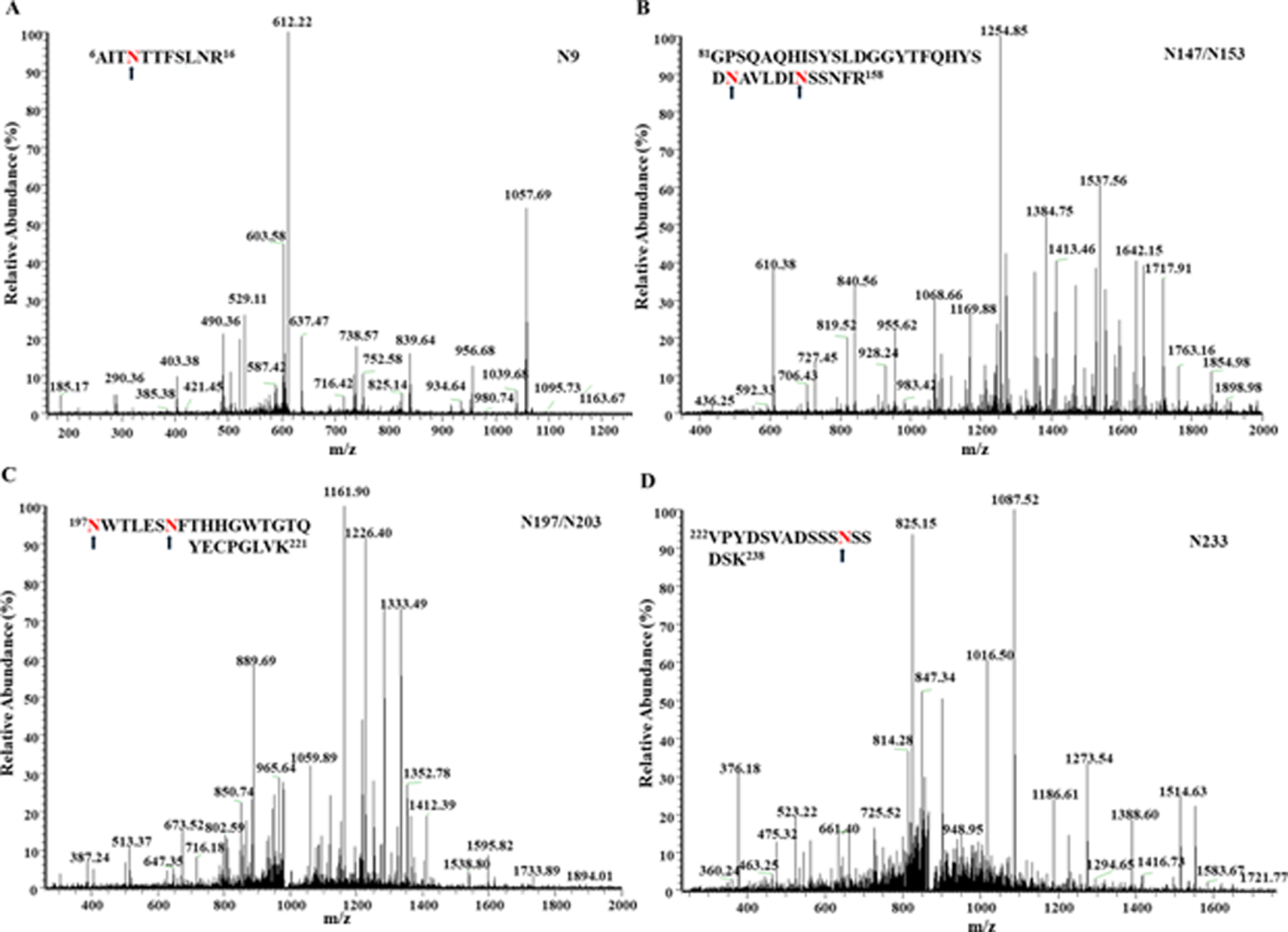


**Fig. S3.** Positive ion ESI-MS analysis of *N*-linked glycan chains for the single-sited mutants of (A) N362Q, (B) N370Q, (C) N399Q, (D) N467Q and (E) N526Q, respectively. The peak of m/z 1905.17 confirmed the presence of the high mannose oligosaccharide (Man)_7_(GlcNAc)_2_. Series of peaks by 162 Da (the mass of anhydrohexose) demonstrated the glycoform heterogeneity. Solid squares and circles represented *N*-acetylglucosamine (GlcNAc) and Mannose (Man), respectively.


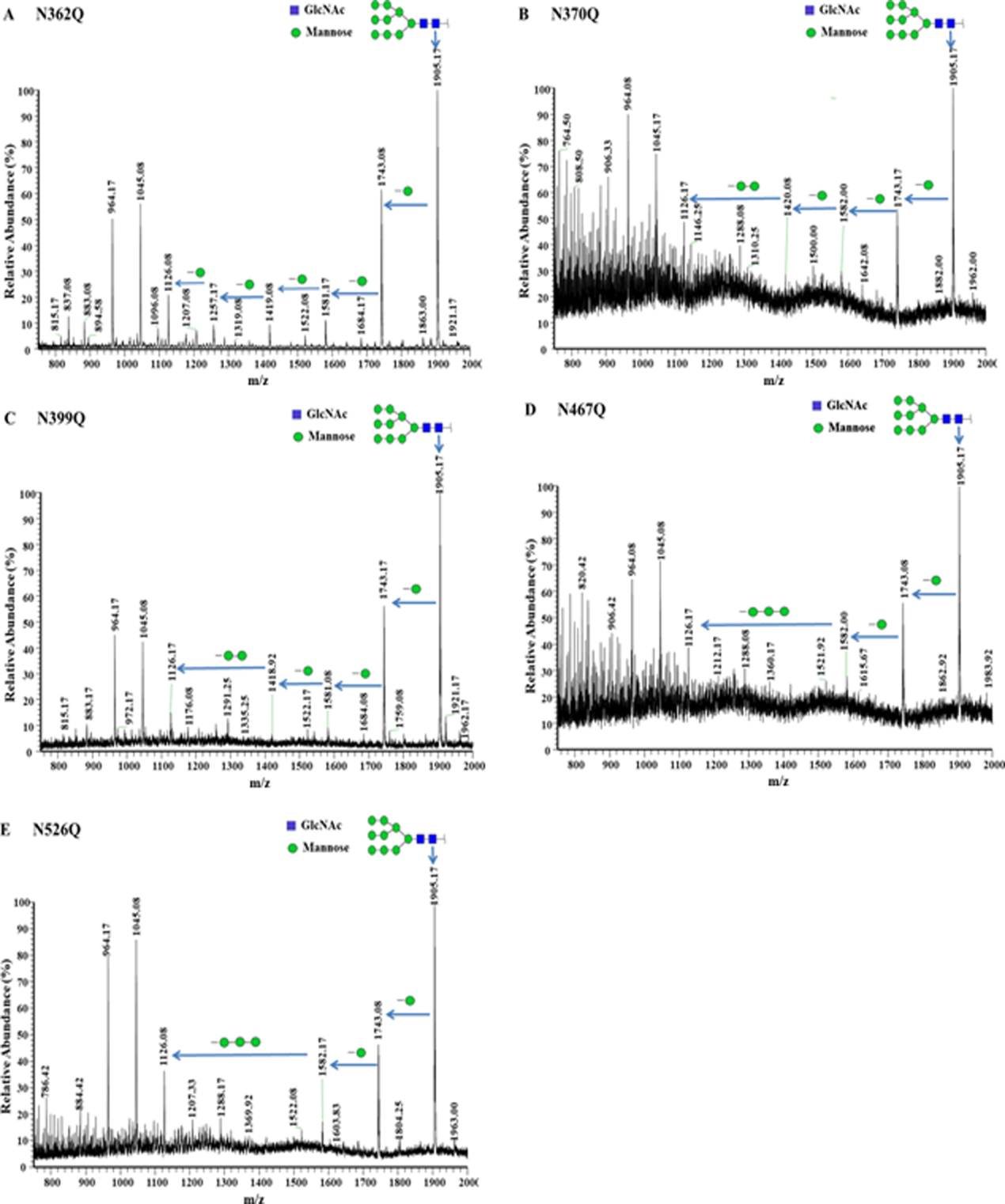


**Table S1** Primers of the site-directed mutagenesis

| Name | Description | Sequence (5’-3’) |
| --- | --- | --- |
| N362Q-F | Mutation at N362 | 5’-CCAAACTTGAAAAACTGGACCTTGGAATCCAACTTCACCCACCACGGC-3’ |
| N362Q-R | Mutation at N362 | 5’-TGTAGTTTGTGATGCTGTAAGTGGTACC***TTG***CTTTCTCAAAGCATCATAGTTC-3’ |
| N370Q-F | Mutation at N370 | 5’-CCAAACTTGAAAAACTGGACCTTGGAATCCAACTTCACCCACCACGGC-3 |
| N370Q-R | Mutation at N370 | 5’-CGTTTTCGGAGGTGACGGTGTA***TTG***TGTGATGCTGTAAGTGGTAC-3’ |
| N399Q-F | Mutation at N399 | 5’-CCAAACTTGAAAAACTGGACCTTGGAATCCAACTTCACCCACCACGGC -3’ |
| N399Q-R | Mutation at N399 | 5’-CGTTGCTCTTGATATCTGGGGAGCC*TTG*AAACACGTATTCAAGATGGAAT-3’ |
| N467Q-F | Mutation at N467 | 5’-TGGCAGTTACCAACCCAGTTTCC***CAA***TACACCACAAACGTCTTCG-3’ |
| N467Q-R | Mutation at N467 | 5’-GCCGCTCAAAGGTTAAATTGGGTAACGTTAAATGAGTTAATGGTGTAAGCC-3’ |
| N526Q-F | Mutation at N526 | 5’-TGGCAGTTACCAACCCAGTTTCCAACTACACCACAAACGTCTTCG-3’ |
| N526Q-R | Mutation at N526 | 5’-GCCGCTCAAAGGTTAAATTGGGTAAC***TTG***AAATGAGTTAATGGTGTAAGCC-3’ |

* The mutation sites were in *italics* and **in bold**.

**Table S2** The enzyme activity of the wild-type rKcINU1 treated with Endo F1

| Digestion time | Sample | Relative activity (%) | |
| --- | --- | --- | --- |
|  |  | Inulin | Sucrose |
| 4 h | Control | 100.00±2.94 | 100.00±3.89^a^ |
|  | Endo F1 | 69.97±5.01 | 62.52±4.17 |
| 16 h | Control | 100.00±1.69 | 100.00±1.78 |
|  | Endo F1 | 36.12±4.24 | 33.95±1.74 |

The value represented an average of three replications. ^a^mean ± SD.
